# ECG-FM: an open electrocardiogram foundation model

**DOI:** 10.1093/jamiaopen/ooaf122

**Published:** 2025-10-16

**Authors:** Kaden McKeen, Sameer Masood, Augustin Toma, Barry Rubin, Bo Wang

**Affiliations:** Toronto General Hospital Research Institute, University Health Network, Toronto, M5G 2C4, Canada; Peter Munk Cardiac Centre, University Health Network, Toronto, M5G 2N2, Canada; UHN AI Hub, University Health Network, Toronto, M5G 2C4, Canada; Vector Institute for Artificial Intelligence, Toronto, M5G 0C6, Canada; Department of Laboratory Medicine and Pathobiology, University of Toronto, Toronto, M5S 3K3, Canada; Toronto General Hospital Research Institute, University Health Network, Toronto, M5G 2C4, Canada; Department of Medicine, University of Toronto, Toronto, M5S 3H2, Canada; Vector Institute for Artificial Intelligence, Toronto, M5G 0C6, Canada; Department of Medical Biophysics, University of Toronto, Toronto, M5G 2C4, Canada; Toronto General Hospital Research Institute, University Health Network, Toronto, M5G 2C4, Canada; Peter Munk Cardiac Centre, University Health Network, Toronto, M5G 2N2, Canada; UHN AI Hub, University Health Network, Toronto, M5G 2C4, Canada; Toronto General Hospital Research Institute, University Health Network, Toronto, M5G 2C4, Canada; Peter Munk Cardiac Centre, University Health Network, Toronto, M5G 2N2, Canada; UHN AI Hub, University Health Network, Toronto, M5G 2C4, Canada; Vector Institute for Artificial Intelligence, Toronto, M5G 0C6, Canada; Department of Laboratory Medicine and Pathobiology, University of Toronto, Toronto, M5S 3K3, Canada; Department of Medical Biophysics, University of Toronto, Toronto, M5G 2C4, Canada; Department of Computer Science, University of Toronto, Toronto, M5S 2E4, Canada

**Keywords:** foundation model, electrocardiography, self-supervised learning, deep learning, time series analysis

## Abstract

**Objectives:**

To develop ECG-FM, an open-weight foundation model for electrocardiogram (ECG) analysis, rigorously evaluate its performance on clinically salient tasks, and openly release it alongside a public benchmark.

**Materials and Methods:**

In a study using 1.5 million 12-lead ECGs, we present ECG-FM, a transformer-based foundation model pretrained with hybrid self-supervision that combines masked reconstruction and contrastive learning with ECG-specific augmentation. Downstream, we evaluate multi-label ECG interpretation and prediction of reduced left ventricular ejection fraction (LVEF), introducing an openly available benchmark on the MIMIC-IV-ECG dataset. We assess ECG-FM’s capabilities through data scaling experiments, latent-space structure analysis, and attention-based saliency.

**Results:**

Finetuned ECG-FM models outperform task-specific baselines in the small-to-medium-scale data regime, exhibit strong label efficiency and cross-dataset generalizability, and achieve high AUROC on salient labels, including atrial fibrillation (0.996) and LVEF ≤40% (0.929). The pretrained encoder showcases competitive linear probing performance, with functionally discriminative embeddings.

**Discussion:**

Findings indicate that ECG-FM is generalizable, label-efficient, and discriminative for screening, risk stratification, and monitoring. Its representations capture low-level morphology and high-order cardiac semantics, and the pretrained encoder serves as a robust feature-set generator. This work mitigates reliance on large labeled datasets, reduces compute and data requirements, and lowers barriers to reproducibility and cross-study comparison.

**Conclusion:**

ECG-FM is an open, rigorously validated ECG foundation model intended to accelerate transparent, comparable research in the ECG analysis subfield. It is designed for rapid integration and evaluation, especially for delivering practical gains in low-label settings. We release our code, model weights, tutorials, and benchmark at https://github.com/bowang-lab/ECG-FM/.

## Introduction

AI-based ECG analysis methods have outperformed traditional computerized interpretation,^1–3^ even matching or exceeding human performance.^4–6^ Recent technological advancements and growing large, publicly available datasets have seen conventional task-specific models replaced by foundation models due to their high performance and reduced reliance on costly labeled data.

Through self-supervised learning (SSL), foundation models are pretrained to encode ECG structure and semantics, then finetuned on downstream tasks with fewer labeled examples. SSL techniques fall into generative or contrastive categories, each with distinct strengths and limitations. Generative methods reconstruct signals from masked inputs,^7–9^ capturing local structural patterns but potentially underrepresenting high-level cardiac semantics.^10^ Contrastive approaches learn discriminative representations from augmented ECG samples,^11–13^ but risk faulty alignment, wherein augmentations degrade physiologically significant patterns.^9^^,^^14^ Hybrid SSL techniques combine both approaches^15^^,^^16^ to capture low-level patterns while preserving semantic information, though augmentation-based strategies remain vulnerable to faulty alignment.

Oh et al^16^ proposed a hybrid approach that circumvents faulty alignment and is based on well-established SSL objectives. They adopted the transformer-based model and generative masking objective from wav2vec 2.0,^17^ as well as the Contrastive Multi-Segment Coding (CMSC) objective, originally introduced in Contrastive Learning of Cardiac Signals (CLOCS).^11^ CMSC treats temporally adjacent ECG segments as positive pairs, exploiting the relative stability of cardiac function over short intervals and eliminating the need for augmentation altogether. Oh et al^16^ also introduced Random Lead Masking (RLM), an ECG-specific augmentation wherein leads are stochastically masked, and demonstrated that by exposing the model to diverse lead combinations during pretraining, their model can be finetuned using arbitrary reduced lead sets of the standard 12-lead ECG.^16^ This leads to a flexible model which prioritizes both low-level patterns and high-level semantic information, while also addressing common drawbacks associated with SSL approaches.

Progress in the ECG analysis field is slowed by unshared weights which hinder reproducibility and comparability. Even when code is released, training modern SSL approaches is often cost-prohibitive. While patient privacy can preclude model release, the availability of large public ECG datasets now makes competitive open-weight pretraining feasible, shifting the barrier from data access to responsible model development and evaluation.

In this study, we present ECG-FM, a transformer-based ECG foundation model pretrained on 1.4 million ECG segments using a hybrid self-supervised objective. ECG-FM is validated on multi-label interpretation and reduced LVEF prediction tasks, demonstrating strong performance, label-efficiency, and generalization. To lower entry costs and enable comparison, we release the weights, code, tutorials, and a public benchmark.

## Methods

### Data

We collected 1.5 million standard 12-lead ECGs from the UHN-ECG, PhysioNet 2021,^18–20^ and MIMIC-IV-ECG^20^^,^^21^ datasets. Specifically, we include 6 datasets in PhysioNet 2021: CPSC, CPSC-Extra, PTB-XL, Georgia, Ningbo, and Chapman. The PTB and St Petersburg INCART datasets are excluded due to having few long samples with inconsistent sampling rates. The MIMIC-IV-ECG v1.0 database contains many 10 s ECGs collected from the Beth Israel Deaconess Medical Center. PhysioNet 2021 and MIMIC-IV-ECG are 2 public data sources used for our pretraining dataset, while UHN-ECG is external to pretraining; we evaluate solely on UHN for downstream tasks, providing evidence of cross-dataset generalization to an institutional cohort.

#### UHN-ECG

UHN-ECG is a newly assembled private, institutional dataset containing 622k ECG recordings from 211k patients who were seen in the emergency department and/or admitted to hospital between January 2010 and December 2018. This 9-year dataset was collected at Toronto General Hospital and Toronto Western Hospital, 2 acute care hospitals with emergency departments, cardiology wards, and coronary care units. These exist as part of the University Health Network (UHN), a network of academic hospitals located in Toronto, Canada. All recordings are 10 s, where 88.8% have an original sampling frequency of 500 Hz and the remaining have 250 Hz. Every ECG has a cardiologist over-read and there are several associated clinical reports and auxiliary data modalities which make for excellent label availability. We report split- and task-specific age and sex in respective Tables S2 and S3. Although 12.8% of ECGs were labeled with poor data quality, we noticed interpretation may be attempted regardless; therefore, we opted to retain these samples and produce a model more tolerant of real-world artifacts.

#### ECG preprocessing

We extracted raw waveforms and tabularized ECG metadata, including sample rates, sample size, and patient demographic information wherever available. We resampled the waveforms at 500 Hz using linear interpolation, performed z-score normalization, and segmented the signals into non-overlapping 5 s segments to produce the model inputs.

### Cohort curation

**Figure 1.**
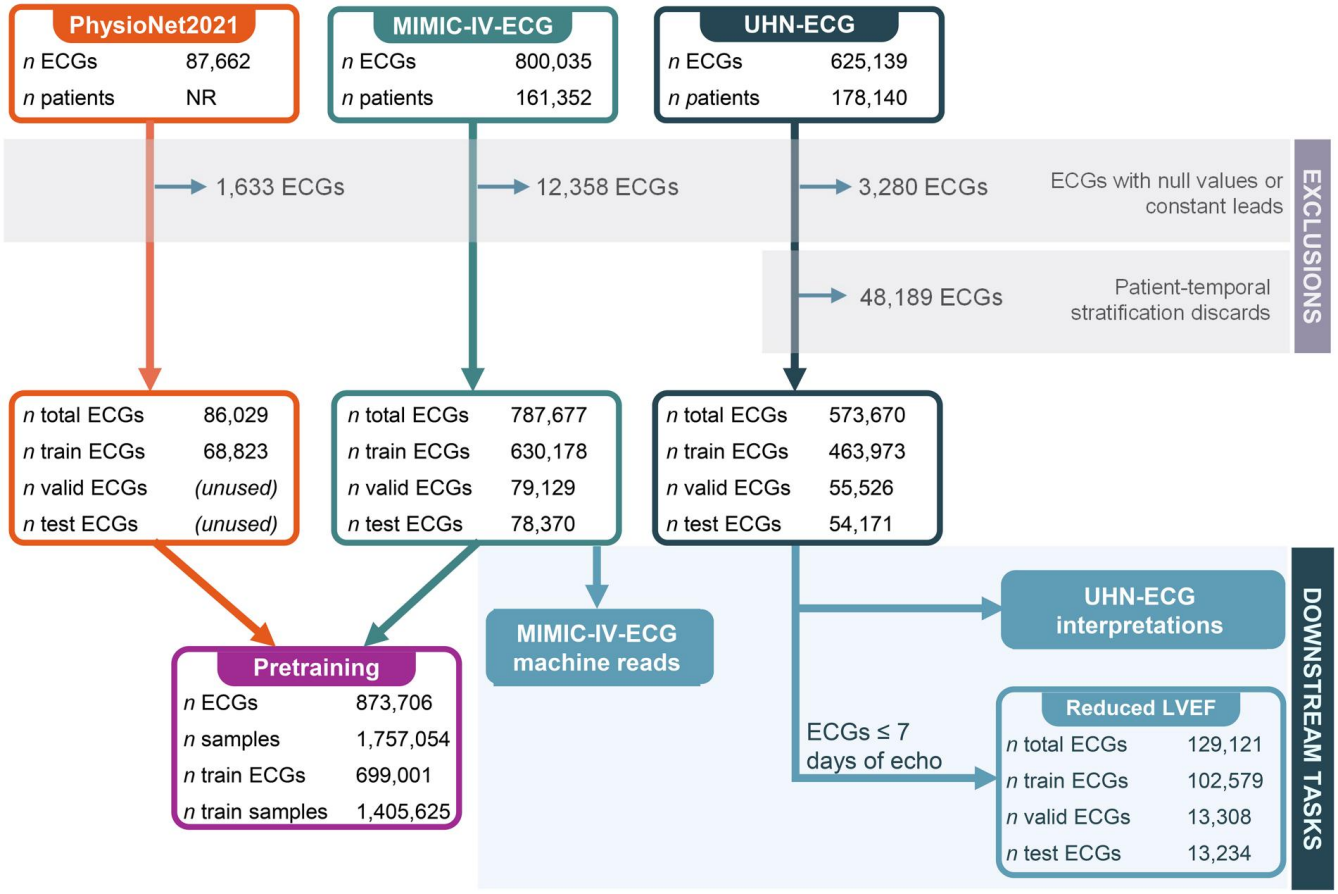
Cohort and sample selection. This flow diagram shows the data sources and ECG exclusion criteria, as well as the dataset partitioning. The pretraining cohort combines samples from public datasets PhysioNet2021 and MIMIC-IV-ECG. Downstream task cohorts utilize MIMIC-IV-ECG and UHN-ECG datasets, where the reduced LVEF task undergoes filtering according to task-specific label availability.

As seen in Figure 1, we removed ECGs with null values or constant-valued leads. To maintain representative distributions and avoid selection bias, no additional exclusions were applied. Each dataset was stratified into 80%/10%/10% splits for training, validation, and testing. UHN-ECG was stratified by patient and temporally to prevent overlap across splits, avoiding label leakage and enabling prospective-like evaluation (see Figure S1). MIMIC-IV-ECG was split by patient only due to imprecise acquisition dates. PhysioNet 2021 was randomly split, with evaluative sets unused. UHN-ECG was excluded from pretraining to respect patient privacy and evaluate cross-dataset generalizability.

### Model architecture

ECG-FM has 90.9 million parameters and uses the wav2vec 2.0 architecture,^17^ consisting of a multi-layer CNN feature extractor and BERT-like transformer encoder. The feature extractor embeds raw signal portions into latent representations $z_{t}$, which feed a transformer encoder to create contextualized representations $c_{t}$.

The feature encoder contains 4 blocks, each with a convolutional layer (256 channels, stride 2, kernel length 2), layer normalization, and a GELU activation. Relative positional embeddings are added to the latent representations. The transformer encoder follows BERT-Base, having 12 layers, 768 embedding dimensions, 12 attention heads, and 3072 feed-forward dimensions.

### Pretraining method

**Figure 2.**
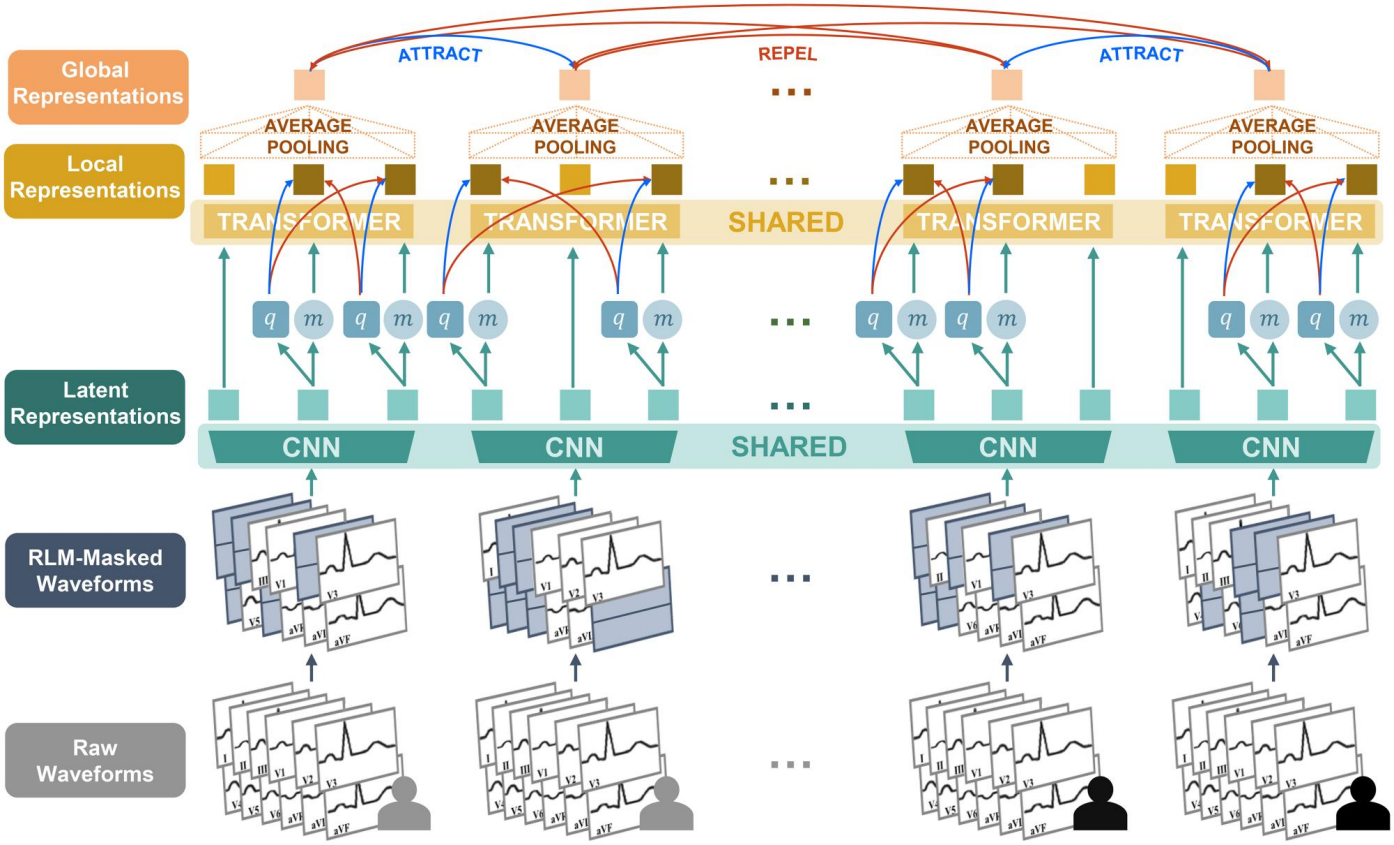
Framework illustration. Raw waveforms are inputted and individual leads are randomly masked. A convolutional feature encoder generates latent representations that feed into a transformer encoder, producing local representations that are then average-pooled to create global representations. Latent representations are randomly masked in spans as *m*, and are quantized to *q*. We then apply a local contrastive loss attracting each *q* to its corresponding local representation, using a subset of other *q* as the negative samples, or distractors. A batch of 4 ECG inputs, making up 2 positive pairs of temporally-adjacent ECG segments, are shown to visualize the CMSC global contrastive loss acting on the global representations across samples. Positive and negative contrastive learning pair relationships are depicted using blue and red arrows, respectively.

We build upon the work of Oh et al,^16^ adopting their pretraining method and engaging in open-source collaboration (https://github.com/Jwoo5/fairseq-signals/). As seen in Figure 2, this hybrid self-supervised approach combines wav2vec 2.0’s continuous signal masking,^17^ CLOCS’s contrastive CMSC objective,^11^ and RLM augmentation.^16^ Previously termed W2V+CMSC+RLM, we refer to this method as WCR.

#### wav2vec 2.0

Inspired by masked language modeling, wav2vec 2.0 masks spans of CNN latent representations $z_{t}$. Each token has 6.5% probability of being a starting index; if selected, we mask 10 subsequent tokens, resulting in approximately 49% masked tokens. We quantize $z_{t}$ to $q_{t}$ using 2 trainable codebooks of 320 codes to remove artifacts that may otherwise trivialize the task.^17^ A contrastive loss maximizes cosine similarity between quantized target $q_{t}$ and corresponding contextualized representation $c_{t}$, while minimizing similarity with distractors $\tilde{q}∼Q_{t}$ sampled from all masked token targets $Q_{t}$.

#### CMSC

CMSC applies contrastive learning between global representations, treating temporally adjacent ECG segments as positive pairs.^11^ This augmentation-free strategy avoids faulty alignment while encouraging consistent representations between consecutive segments, promoting temporal invariance and capturing functionally relevant information over superficial differences.

#### RLM

RLM masks each lead with probability P=0.5, enhancing robustness through diverse lead combinations during pretraining.^16^ Although not explored in this work, RLM enables finetuning on arbitrary lead subsets, making ECG-FM applicable in contexts where only reduced 12-lead sets are available.

### Downstream tasks

To demonstrate that ECG-FM is useful for a variety of downstream applications, we selected several classification tasks for our evaluation. For each task, we report split-specific outcome prevalence in Table S4 and show receiver operating characteristic (ROC) curves and the precision-recall curves (PRC) for each label in our *Full*, *Random Init.*, and *Linear* experiment suites, reporting the label names in the legend.

#### UHN-ECG interpretation

As primary readers, cardiologists demonstrate greater interpretation accuracy and less interobserver variability compared to physicians with less specialized training.^22^^,^^23^ In the UHN-ECG dataset, each ECG is accompanied by a cardiologist’s interpretation, provided as an over-read of an automated analysis. We extracted binary labels from these free-text expert interpretations using a knowledge graph and text-parsing system. Further details can be found in Section S1.1.

#### MIMIC-IV-ECG machine reads

MIMIC-IV-ECG utilizes machine measurements to automatically generate ECG reports. For this task, we generate our labels from these reports using the same text-parsing system as with the UHN-ECG interpretation task, adjusting the patterns to accommodate dataset-specific terminology. We focused on all the same labels as in the UHN-ECG interpretation task, given they were available. Our conversion of free-text interpretations into binary labels culminates in an accessible benchmark task.

#### UHN-ECG reduced LVEF

Heart failure is a major contributor of morbidity and mortality worldwide, with approximately 50% of cases being heart failure with reduced ejection fraction (HFrEF).^24^ The heightened risk of mortality associated with HFrEF, even in asymptomatic cases, underscores the significance of early detection for timely intervention.^25^^,^^26^ We predict low, or reduced, LVEF as a step towards early screening for left ventricular systolic dysfunction and HFrEF. Using regex to extract LVEF percentages from echocardiography reports, we generated labels at various common LVEF thresholds: ≤50%, ≤40%, ≤35%, and ≤30%. We paired each ECG with its closest associated echocardiography report which indicates an LVEF percentage, taking only those ECG samples with a valid report within ±7 days of acquisition.

### Experiments

The ECG-FM model was pretrained on 1 405 625 samples using 3 A100 80GB GPUs applying distributed data parallelism. The 1026 batch size was composed of 171 positive pairs, each consisting of 2 segments, distributed across the 3 GPUs. A fixed learning rate schedule was used, where it was initially set at $1\times 10^{-4}$ for the first 5 epochs, reduced to $8\times 10^{-5}$ from epoch 6 to 200, and further decreased to $5\times 10^{-5}$ for epochs 201 to 240. Training concluded after 240 epochs, totaling a computational wall time of 9.51 days. We do not perform ablation studies on WCR, as these have been performed previously by Oh et al.^16^

We ran several suites of ECG-FM multi-label classification experiments for each downstream task. Loss balancing was applied using weights inversely proportional to the outcome prevalence. We initialized our *Full* models with the pretrained weights and performed full finetuning, wherein all model weights are updated, using a learning rate of $1\times 10^{-6}$. For *Random Init.*, we randomly initialized the models and then performed full finetuning with a learning rate of $1\times 10^{-5}$. To maintain a fair comparison, the weight initialization and learning rates were the only experimental differences between the *Full* and *Random Init.* experiments. In the *Linear* experiment, we performed linear probing with learning rate $1\times 10^{-5}$. For this frozen evaluation, pretrained model embeddings are extracted and fed as inputs to a single linear layer to generate predictions.

We also employed 2 competitive, task-specific baseline models: The *Nejedly* baseline is a ResNet with multi-head attention which follows the configuration seen in Nejedly et al^27^; the *SE-WRN* baseline implementation is similar to that of Han et al.^28^ Each is trained with a learning rate of $1\times 10^{-4}$.

Data scaling experiments were performed by taking a percentage subset of ECGs in the training set while maintaining the same evaluative sets. We ran experiments on 50%, 10%, and 1% of the training set ECGs for the interpretation tasks, as well as 50% and 10% for the reduced LVEF task.

All downstream tasks ran on a single A100 80GB GPU using a batch size of 256. Experiments used the Adam optimizer^29^ with $\beta_{1}=0.9$, $\beta_{2}=0.98$. Checkpoints were selected according to which had the best AUPRC on the validation set. Thresholds were computed to achieve target recall values on the validation set. Aside from the aggregated results shown in Table S5, all evaluative metrics are computed by randomly sampling a single segment per ECG.

We mitigate class imbalance by applying a per-label positive-class reweighting scheme during finetuning using a standard $\text{pos}\_{\text{weight}}_{k}=\frac{N_{\text{neg},k}}{N_{\text{pos},k}}$ per label *k*, as computed from the training split sample counts. We report Area Under the Precision-Recall-Gain curve (AUPRG)^30^ to support prevalence-robust evaluation (see Section S1.3).

## Results

### Data scaling

#### Pretraining benefits

**Figure 3.**
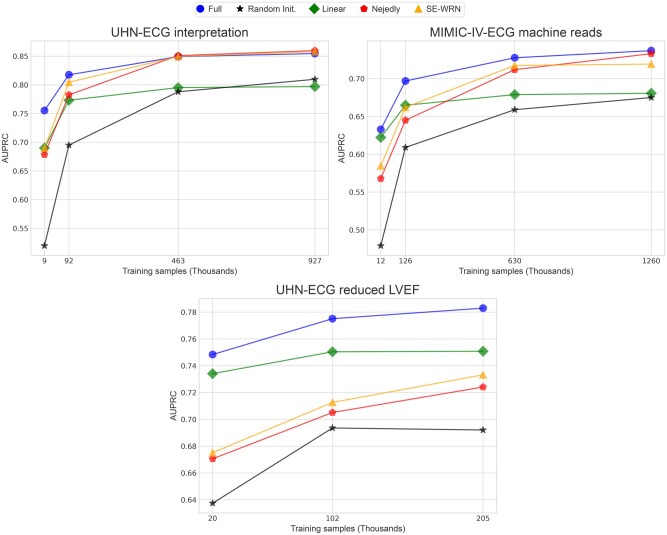
Data scaling results. Label-averaged AUPRC across experiment suites and training dataset sizes for all tasks.

The *Linear* results in Figure 3 demonstrate that our pretrained model embeddings encode rich, task-relevant information. The *Random Init.* performance is relatively poor with few training samples, confirming that ECG-FM’s pretraining is responsible for its superior data efficiency. At the smallest training set sizes, *Linear* outperforms the baselines and performs comparably to *Full* in the MIMIC-IV-ECG machine reads and UHN-ECG reduced LVEF tasks; however, its performance plateaus because it lacks the representational capacity necessary to exploit additional downstream data. Although task-dependent, this indicates that the benefits of WCR pretraining are quite significant in smaller data regimes and transfer well to unseen task-specific datasets.

#### Downstream performance

Across all tasks, *Full* outperforms the baselines given approximately 100 000 training samples. However, for the UHN-ECG interpretation and MIMIC-IV-ECG machine reads tasks, the *Full* and baseline model performances trend to a similar plateau. ECG-FM tends to outperform strong task-specific models on downstream tasks, especially in the small-to-medium-scale data regime; however, it may not provide significant value downstream given sufficiently large task-specific datasets.

### Latent space analysis

**Figure 4.**
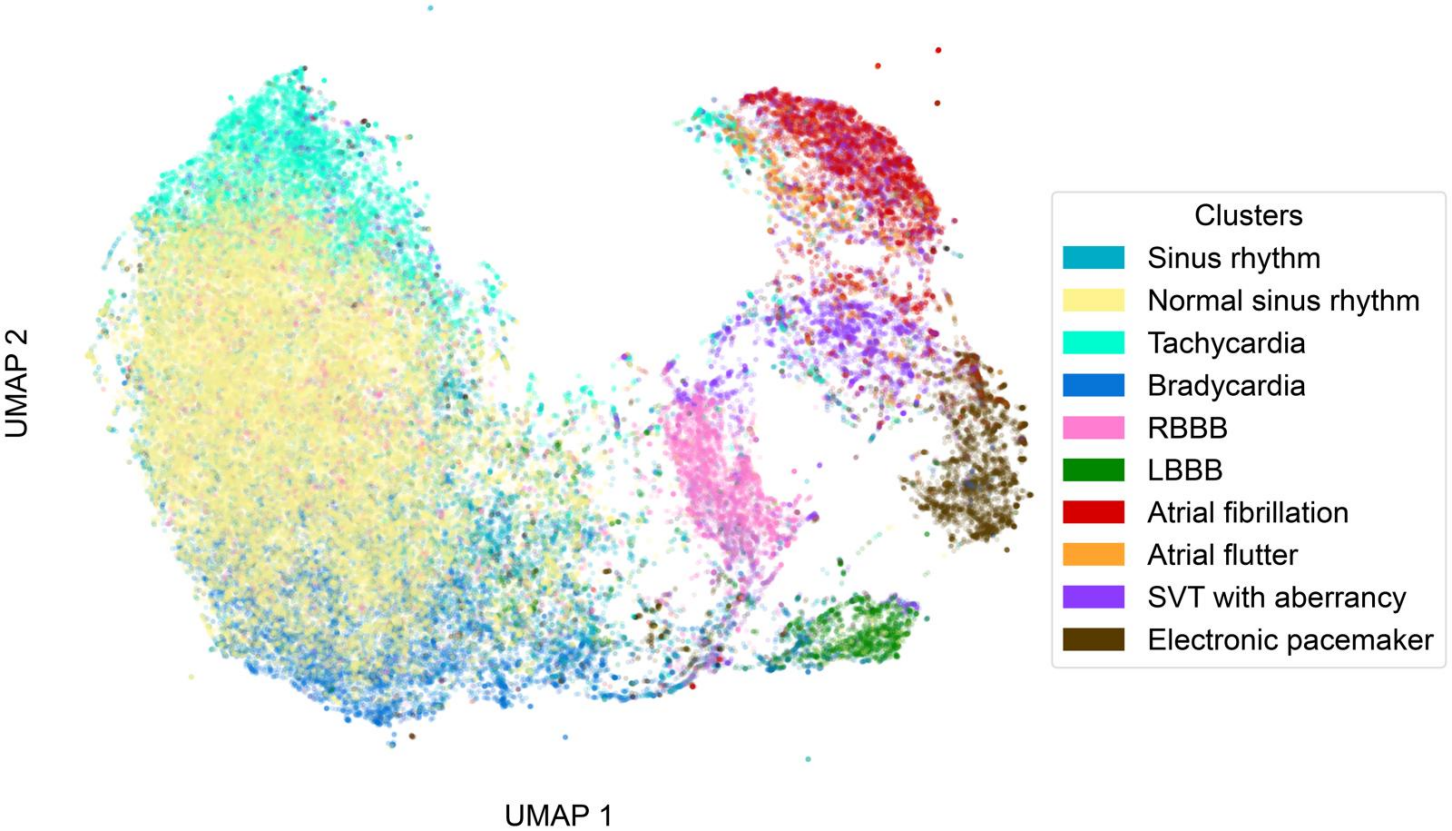
Pretrained latent space UMAP. A UMAP visualization of pretrained ECG-FM global representations from ECGs in the UHN-ECG dataset. An additive color scheme employs select labels from the UHN-ECG interpretation task to enable a latent space analysis, wherein some prioritization was performed to prevent label overlap from reducing readability.

The UMAP visualization of the UHN-ECG dataset in Figure 4 highlights ECG-FM’s ability to encode relationships in rhythm, heart rate, and pathology for an unseen dataset. This latent space depicts our pretrained model—which has never been trained on any labels—making the label overlay strictly evaluative. Additional label-specific UMAP views are provided in Figure S2.

A distinct *Normal sinus rhythm* cluster sits within a broader *Sinus rhythm* region, with *Bradycardia* and *Tachycardia* samples forming its extremities and encoding heart rate along the vertical axis. Non-sinus tachycardias like *Atrial fibrillation* and *Atrial flutter* occupy a separate upper region, while conduction defects, such as the bundle branch blocks, cluster more centrally. This spatial arrangement, along with the close proximity of related tachycardias, suggests ECG-FM captures functional similarity. A pacemaker-positive evaluative subset revealed no performance drop for other labels despite the strong clustering of electronic pacemaker samples, indicating a robust encoding of diverse relationships beyond those visually evident in Figure 4. Overall, these observations highlight ECG-FM’s capacity to produce physiologically meaningful and functionally discriminative representations.

### Downstream tasks

#### UHN-ECG interpretation

As evidenced in Table S7, we achieve strong performance across numerous labels using a cohort representative of real in-hospital populations. Our results demonstrate that ECG-FM is resilient even to low-quality recordings, with a considerable 12.4% of the test set labeled with *Poor data quality*. Per-label AUPRG (see Section S1.3) is generally quite high across labels and confirms reliable performance even for extremely rare labels. We report ROC and PRC curves in Figure S3.

#### MIMIC-IV-ECG machine reads

To evaluate the quality of this task for benchmarking, we compare Tables S6 and S8 with labels shared with the UHN-ECG interpretation task. While AUPRC is often lower on this task than their UHN-ECG equivalent, re-expressing performance with AUPRG narrows cross-dataset differences and yields several labels with comparable performances. Residual discrepancies concentrate in labels whose annotation likely benefits from cardiologist adjudication, consistent with differences in label sourcing. This indicates that the machine read labels rely on patterns consistently recognizable by ECG-FM, supporting this task as a practical benchmark, while highlighting performance discrepancies that are plausibly attributable to label quality rather than model limitations. We report ROC and PRC curves in Figure S4.

#### UHN-ECG reduced LVEF

Table S9 presents performances across all experiment suites, where our *Full* experiment outperforms both baselines across all data scales and labels. It is unclear whether the performance gap between the baselines and *Full* would similarly close, as with the other tasks, given greater task-specific data availability. We see an upwards trend in the *Full* experiment performance which suggests that WCR pretraining would continue to provide benefit given a larger dataset. We report ROC and PRC curves in Figure S5.

### Saliency maps

**Figure 5.**
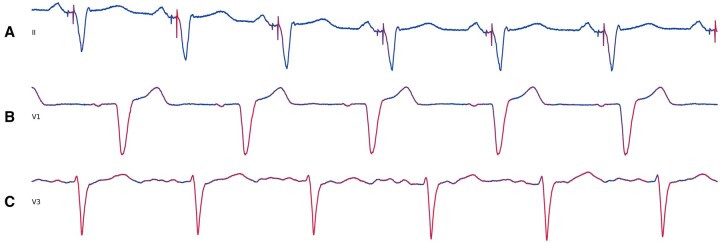
Saliency maps. Distinct 5 s ECG segments colored using corresponding self-attention weight activations derived from pretrained, full-finetuned ECG-FM models. Red represents a higher relative activation. (A) UHN-ECG interpretation model activations for an ECG labeled with ventricular pacing (lead II); (B) UHN-ECG interpretation model activations for an ECG labeled with LBBB (lead V1); and (C) UHN-ECG reduced LVEF model activations for an ECG labeled with LVEF≤30% (lead V3).

We generated attention-based saliency maps by extracting attention weights from the final transformer encoder self-attention layer, averaging across attention heads, and projecting these into the input space. The visualizations in Figure 5 serve as a heuristic for relative input importance. Our models consistently attend to relevant regions across cardiac cycles. For instance, in predicting *Ventricular pacing*, attention focuses on pacing spikes—a hallmark of paced rhythms—indicating sensitivity to focal, clinically relevant patterns. This preferential attention suggests effective use of local contextual information for prediction.

## Discussion

We demonstrate strong performance of ECG-FM on clinically relevant tasks. The UHN-ECG interpretation experiment marks a step toward full, expert-level 12-lead interpretation, while the reduced LVEF task highlights ECG-FM’s potential to inform rapid development of medical management plans. Evaluated in a simulated prospective setting that reflects representative in-hospital populations and tracing quality, these UHN-ECG tasks provide strong evidence for ECG-FM’s cross-dataset generalizability.

Standard metrics can be misleading under class imbalance: Metrics like accuracy are dominated by the majority class and AUPRC shifts with outcome prevalence. The depressed AUPRC observed for rare outcomes partly reflects low positive rates rather than poor discrimination, as per-label AUPRG remains high in our prevalence-robust evaluation and better reflects model utility (Section S1.3).^30^ Furthermore, this analysis indicates that performance primarily tracks ECG signature specificity and label ontology, offering a promising explanation for the lower relative performances of more composite labels *Myocardial infarction* and *Poor data quality*.

Our data scaling experiments demonstrate rapid adaptability to downstream tasks and reduced reliance on labeled data. ECG-FM consistently outperforms conventional task-specific methods in the small-to-medium-scale data regime, underscoring the benefit of WCR pretraining. The linear probing experiments confirm that the pretrained embeddings encode task-relevant information, suggesting that ECG-FM can act as a robust, competitive feature-set generator for diverse clinical applications.

Our results suggest that ECG-FM commands local and global contextual information effectively. In our saliency maps, it displays relevant, preferential attention to the same regions across the cardiac cycle. Our latent space analysis exhibits pretrained embeddings which are functionally discriminative and indicative of ECG-FM’s ability to capture underlying cardiac function. Such explorations form a basis to demystify internal model workings and improve model interpretability.

CMSC’s positive pair strategy requires 2 consecutive segments, necessitating ECG-FM accept 5 s ECG inputs rather than the full 10 s available in the MIMIC-IV-ECG and UHN-ECG datasets. Our labels are not segment-specific, creating an input-to-label mismatch in the case of focal patterns which are more present in 1 segment than its neighbor. We perform a segment-aware evaluation in Section S1.2 which significantly improves performance on select labels, showcasing that these effects are mitigable. As discussed in Section S1.3, one limitation is the label-space rigidity that results from predicting a fixed set of binary labels. We partially mitigate this with a curated knowledge graph that captures and aggregates condition subtypes (see Section S1.1); however, any captured nuance in clinician interpretation is then collapsed into composite labels which conflate heterogeneous phenotypes. Text-based methodology or hierarchical labeling may better capture such granularity in future work. Other promising avenues for extending this work include integration of ECG-FM into text-conditioned frameworks and multimodal foundation models as an ECG encoder.

## Conclusion

We present ECG-FM, an open-weight ECG foundation model pretrained using a hybrid SSL method and evaluate it across clinically salient tasks. Our results indicate that ECG-FM is a robust, generalizable, and functionally discriminative model which performs strongly in low-label regimes, alleviating the need for large annotated datasets. By releasing the model, code, tutorials, and a public benchmark, we aim to accelerate transparent, comparable research and streamline practical adoption—especially where labeled data and compute are constrained.

## Supplementary Material

ooaf122_Supplementary_Data

## Data Availability

PhysioNet 2021 v1.0.3 is available for public download (https://doi.org/10.13026/34va-7q14), as is MIMIC-IV-ECG v1.0 (https://doi.org/10.13026/4nqg-sb35). The UHN-ECG dataset is not available for public use. Model weights are available on our GitHub for our pretrained model and downstream MIMIC-IV-ECG machine reads models. Downstream UHN-ECG models cannot be made available due to privacy concerns. Code for data preprocessing, model training, model inference, and experiment reproduction is available, as are tutorial notebooks. Refer to https://github.com/bowang-lab/ECG-FM/.
