# Supplementary material for: ECG-FM: an open electrocardiogram foundation model
Source: JAMIA Open. 2025 Oct 16;8(5):ooaf122. doi: 10.1093/jamiaopen/ooaf122 (PMC12530324; doi:10.1093/jamiaopen/ooaf122)
Supplement: ooaf122_Supplementary_Data [file ooaf122_supplementary_data.zip › Supplementary.pdf]

S1 Supplementary Material

Table S1. Abbreviations.

ECG	Electrocardiogram
UHN	University Health Network
WCR	W2V+CMSC+RLM
W2V	wav2vec 2.0
CLOCS	Contrastive Learning Of Cardiac Signals
CMSC	Contrastive Multi-Segment Coding
RLM	Random lead masking
HFrEF	Heart failure with reduced ejection fraction
LVEF	Left ventricular ejection fraction
PVC	Premature ventricular contraction
SVT	Supraventricular tachycardia
AP	Accessory pathway
AV	Atrioventricular
RBBB	Right bundle branch block
LBBB	Left bundle branch block

Table S2. Biological age. Shown is the Mean ± STD of biological age in years across UHN-ECG's downstream task manifests.

**Split**	**Interpretations**	**Reduced LVEF**
**Train**	62.2 ± 19.5	65.9 ± 16.8
**Valid**	60.8 ± 19.3	66.3 ± 16.1
**Test**	59.8 ± 19.4	65.6 ± 16.5

Table S3. Biological sex. Shown is distribution of sex (% Female) across UHN-ECG's downstream task manifests.

**Split**	**Interpretations**	**Reduced LVEF**
**Train**	45.7%	37.3%
**Valid**	45.9%	39.6%
**Test**	44.9%	40.2%

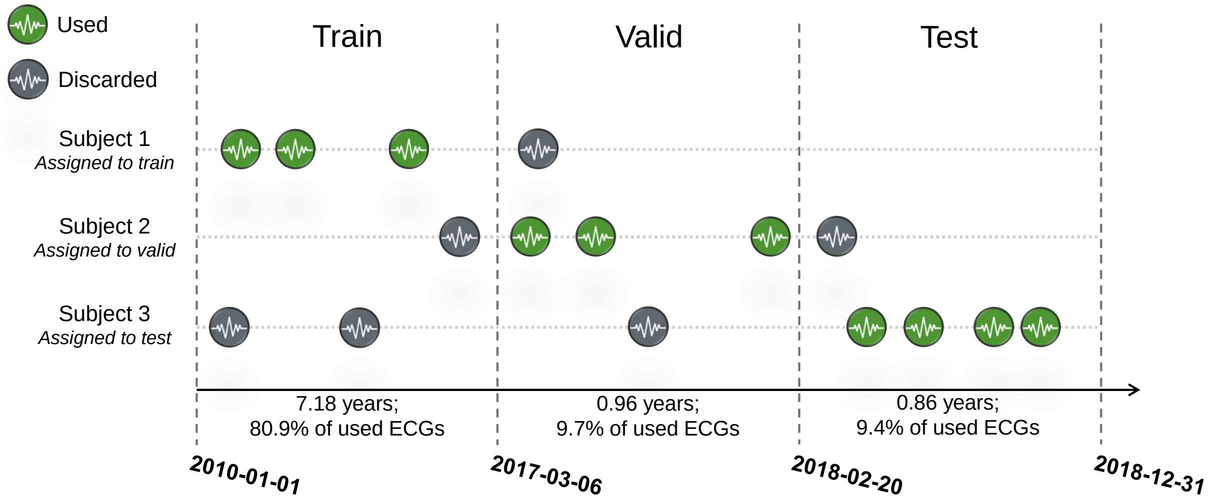

Fig. S1. Illustration of UHN-ECG patient-temporal splits. UHN-ECG dataset splits have no overlap temporally, where temporal cutoffs were estimated to generate an approximate 80/10/10 split. Subjects were assigned to whichever split contained the majority of their ECGs. Patient overlap is not permitted between splits to prevent any possibility of label leakage.

Table S4. Outcome prevalence. Positive label frequency rates (%), as separated by split and task.

Label	Train	Validation	Test
**UHN-ECG interpretations**			
**Poor data quality**	0.126	0.151	0.124
**Sinus rhythm**	0.814	0.817	0.823
**Normal sinus rhythm**	0.465	0.467	0.472
**PVC**	0.064	0.070	0.066
**Tachycardia**	0.218	0.220	0.214
**Ventricular tachycardia**	0.001	0.001	0.001
**SVT with aberrancy**	0.055	0.057	0.049
**Atrial fibrillation**	0.094	0.089	0.084
**Atrial flutter**	0.022	0.024	0.022
**Bradycardia**	0.120	0.120	0.124
**Accessory pathway conduction**	0.115	0.112	0.106
**AV block**	0.093	0.095	0.096
**1st degree AV block**	0.066	0.066	0.070
**2nd degree AV block**	0.024	0.025	0.023
**Bifascicular block**	0.018	0.016	0.019
**RBBB**	0.104	0.107	0.108
**LBBB**	0.038	0.039	0.034
**Myocardial infarction**	0.193	0.187	0.199
**Electronic pacemaker**	0.067	0.063	0.062
**Ventricular pacing**	0.047	0.048	0.046
**Atrial pacing**	0.020	0.015	0.016
**MIMIC-IV-ECG machine reads**			
**Poor data quality**	0.026	0.027	0.026
**Sinus rhythm**	0.812	0.811	0.809
**PVC**	0.065	0.065	0.067
**Tachycardia**	0.209	0.210	0.210
**Ventricular tachycardia**	0.001	0.001	0.001
**SVT with aberrancy**	0.042	0.043	0.040
**Atrial fibrillation**	0.101	0.103	0.103
**Atrial flutter**	0.018	0.019	0.018
**Bradycardia**	0.130	0.132	0.132
**Accessory pathway conduction**	0.119	0.123	0.120
**AV block**	0.078	0.085	0.081
**1st degree AV block**	0.073	0.079	0.076
**Bifascicular block**	0.030	0.033	0.030
**RBBB**	0.082	0.088	0.081
**LBBB**	0.037	0.039	0.038
**Myocardial infarction**	0.224	0.226	0.225
**Electronic pacemaker**	0.039	0.036	0.042
**UHN-ECG reduced LVEF**			
**LVEF≤30%**	0.245	0.141	0.156
**LVEF≤35%**	0.262	0.202	0.214
**LVEF≤40%**	0.345	0.273	0.263
**LVEF≤50%**	0.488	0.420	0.394

S1.1 Interpretation text parsing

In clinical care settings, ECG interpretations are often recorded as free-text. While this approach is convenient and allows for unbounded precision, it can be challenging to translate this unstructured format into precise labels which can be digested by AI models. Synonyms, acronyms, grammar, typographical errors, evolving medical terminology, and implied findings are all examples of complexities which, if not handled with care, can severely lessen label quality, which in turn reduces model effectiveness and evaluative correctness.

Maintaining positional information, we applied pattern matching which was manually curated to parse free-text and match over 99% of UHN-ECG interpretations completely. Derived from these patterns are a series of entities (e.g., 'tachycardia', 'infarction'), descriptors (e.g., 'probably', 'moderate', 'acute'), and connectives (e.g., 'associated with', 'transitions to'). Relevant information from the descriptors and connectives are distilled down into their corresponding entities. We map the resulting entities into labels which can be flexibly manipulated. Using clinician-in-the-loop decision making, we constructed a knowledge graph encoding label relationships which are true by definition. We used it to recursively mark labels as true, for example, labeling *Ventricular tachycardia* when *Torsades de Pointes*, one form of polymorphic ventricular tachycardia, was specifically stated. Without this component, we suspect that the model would learn physiologically arbitrary distinctions which would prove counterproductive to accurate interpretation.

S1.2 Segment-aware evaluation

Our interpretation tasks rely on annotated 10 s recordings, however our model evaluates each recording by cropping it into two non-overlapping 5 s segments, which become inputs utilizing the same label. This cropping is necessary to generate positive pairs for the CMSC contrastive objective. However, it means that we are predicting labels given partial information, where certain diagnoses may not even present in a given segment. To investigate how this affects our results, we explored aggregating model logits by taking the maximum, mean, and minimum for each two adjacent segments.

Label-specific aggregation methods are selected according to which yields the highest AUPRC on the validation set, where the best-performing methods tend to follow a common schema. Labels with 'max' are typically diagnosed using distinct features or have focal morphology such as pacemaker spikes, ectopic beats, and artifacts. Aggregation may help here considerably when such markers are less discernible, or perhaps absent, in one of the segments. The 'mean' labels generally represent more continuous patterns such as rhythms and abnormalities resulting in a sustained change in presentation. Gains with this method are generally more modest and may be acting as an ensemble prediction which benefits from more complete information. Only *Normal sinus rhythm* used the 'min' method, which is reasonable since this condition would not be stated unless both segments met the necessary criteria.

This aggregation experiment crudely quantifies how the model might have performed having seen the full ECG sample. The aggregated metrics may serve as a more accurate depiction of ECG-FM's capabilities, clarifying that certain poor performance is not due to inherent shortcomings as an encoder, but rather to an evaluative limitation. Although we find this aggregation methodology to be reasonable, we refrain from adopting it in the main paper to avoid over-complicating our method.

Table S5. Segment-aggregated UHN-ECG interpretation results. Segment-aggregated test results for *Full*. Percentages indicate metric increases over the non-aggregated results.

Label	Method	AUROC		AUPRC	
**Poor data quality**	max	0.924	(+2.69%)	0.694	(+5.54%)
**Sinus rhythm**	mean	0.993	(+0.07%)	0.998	(+0.03%)
**Normal sinus rhythm**	min	0.987	(+0.87%)	0.985	(+1.52%)
**PVC**	max	0.987	(+4.61%)	0.877	(+16.65%)
**Tachycardia**	mean	0.997	(+0.08%)	0.986	(+0.26%)
**Ventricular tachycardia**	max	0.997	(+0.43%)	0.465	(+2.68%)
**SVT with aberrancy**	max	0.987	(+0.4%)	0.835	(+3.39%)
**Atrial fibrillation**	mean	0.997	(+0.06%)	0.973	(+0.51%)
**Atrial flutter**	mean	0.987	(+0.07%)	0.774	(+1.77%)
**Bradycardia**	mean	0.997	(+0.2%)	0.982	(+1.22%)
**Accessory pathway conduction**	mean	0.996	(+0.06%)	0.976	(+0.34%)
**AV block**	mean	0.988	(+0.12%)	0.915	(+0.71%)
**1st degree AV block**	mean	0.993	(+0.1%)	0.928	(+0.84%)
**2nd degree AV block**	mean	0.987	(+0.14%)	0.734	(+2.18%)
**Bifascicular block**	mean	0.991	(+0.05%)	0.704	(+2.26%)
**RBBB**	mean	0.992	(+0.07%)	0.942	(+0.33%)
**LBBB**	mean	0.993	(+0.06%)	0.869	(+0.5%)
**Myocardial infarction**	mean	0.941	(+0.25%)	0.825	(+0.64%)
**Electronic pacemaker**	max	0.991	(+0.81%)	0.948	(+1.86%)
**Ventricular pacing**	max	0.998	(+0.16%)	0.977	(+0.78%)
**Atrial pacing**	max	0.996	(+0.06%)	0.929	(+0.91%)

S1.3 Prevalence-robust evaluation

Outcome prevalence varies widely in multilabel ECG classification. This makes it difficult to contrast performance across labels using metrics with label-specific performance scales such as AUPRC, where AUPRC's random classifier baseline performance is equal to a label's unique positive sample rate. Area Under the Precision–Recall–Gain curve (AUPRG) enables prevalence-robust evaluation, where these curves re-parameterize the precision-recall space such that a random classifier score maps to zero and a perfect classifier to one. This yields a comparable 0–1 performance scale independent of class balance, thus providing a more accurate depiction of rare-label performance and enabling more concrete comparisons across labels, tasks, and studies.

Per-label AUPRG (see Supplementary Table S6) is generally high, indicating reliable ranking of positives above negatives even under extreme class imbalance. Notably, several rare labels—*Ventricular tachycardia*, *Atrial flutter*, and *LBBB*—achieve near-ceiling AUPRG, showing that ECG-FM performance is not dependent on high outcome frequencies. Common rhythms (*Sinus rhythm*, *Normal sinus rhythm*) and conduction abnormalities (*RBBB*, *LBBB*, *AV block*) show similarly strong gains, while reduced LVEF performance remains high, underscoring sensitivity across structural, conduction, and rhythm conditions. Together, these findings support ECG-FM's robustness and prevalence-independent discriminative ability across heterogeneous ECG phenomena.

We find that performance largely tracks ECG signature specificity and label ontology. Narrow, pathognomonic morphologies with well-defined criteria—e.g., *Atrial fibrillation*, *LBBB*, *RBBB*, *Ventricular tachycardia*—approach ceiling AUPRG across data scales. In contrast, broader or composite constructs—e.g., *Myocardial infarction* (acuity/location heterogeneity) and *Poor data quality* (multiple artifact subclasses)—show lower and more variable AUPRG, including the only < 0.9 AUPRG scores in the UHN-ECG interpretation labels at the 1% scale. This is suggestive of annotation noise exacerbated by pronounced phenotypic heterogeneity. Our labeling system was designed to respect underlying label ontology and phenotypic variance by capturing hierarchical label taxonomy with descriptive subtypes; however, collapsing these into a few binary labels nonetheless resulted in several suboptimal composite labels.

Table S6. Area Under the Precision-Recall-Gain curve (AUPRG) results for all *Full* experiments. Label-specific test AUPRG scores on our pretrained, full-finetuned models for all tasks at different percentages of the full finetuning training set sizes.

**Finetuning train set size (% of total)**	**1%**	**10%**	**50%**	**100%**
**UHN-ECG interpretations**				
**Poor data quality**	0.89885	0.937870	0.951520	0.956280
**Sinus rhythm**	0.91481	0.949280	0.962060	0.965000
**Normal sinus rhythm**	0.93059	0.954580	0.962840	0.965610
**PVC**	0.98084	0.987860	0.990730	0.991680
**Tachycardia**	0.99262	0.995810	0.997210	0.997480
**Ventricular tachycardia**	0.99995	0.999980	0.994910	0.994900
**SVT with aberrancy**	0.98659	0.995280	0.996930	0.997350
**Atrial fibrillation**	0.99815	0.998930	0.999260	0.999310
**Atrial flutter**	0.99704	0.998750	0.999200	0.999330
**Bradycardia**	0.99486	0.997850	0.998450	0.998560
**Accessory pathway conduction**	0.99723	0.998160	0.999010	0.999180
**AV block**	0.98758	0.994230	0.996370	0.996640
**1st degree AV block**	0.99471	0.997730	0.998430	0.998510
**2nd degree AV block**	0.99472	0.996020	0.997940	0.998040
**Bifascicular block**	0.99345	0.994750	0.998450	0.998350
**RBBB**	0.99345	0.996060	0.997140	0.997610
**LBBB**	0.99756	0.999060	0.999340	0.999330
**Myocardial infarction**	0.85887	0.936960	0.957570	0.962910
**Electronic pacemaker**	0.9966	0.998150	0.998700	0.998720
**Ventricular pacing**	0.99887	0.998680	0.999170	0.999580
**Atrial pacing**	0.99905	0.998070	0.998760	0.998630
**MIMIC-IV-ECG machine reads**				
**Poor data quality**	0.87687	0.951400	0.966300	0.969760
**Sinus rhythm**	0.74094	0.835400	0.868950	0.882700
**PVC**	0.95444	0.965870	0.974240	0.977590
**Tachycardia**	0.96842	0.976090	0.978280	0.979360
**Ventricular tachycardia**	0.98861	0.992690	0.999850	0.999890
**SVT with aberrancy**	0.98394	0.989700	0.992960	0.993060
**Atrial fibrillation**	0.98618	0.989910	0.991970	0.992660
**Atrial flutter**	0.98689	0.988450	0.994130	0.994490
**Bradycardia**	0.97391	0.980550	0.983010	0.984080
**Accessory pathway conduction**	0.98488	0.987750	0.989890	0.990800
**AV block**	0.98449	0.989650	0.991310	0.991440
**1st degree AV block**	0.98664	0.990660	0.992300	0.992500
**Bifascicular block**	0.99325	0.996740	0.997450	0.996860
**RBBB**	0.99188	0.995140	0.996400	0.996300
**LBBB**	0.99717	0.997670	0.997550	0.997840
**Myocardial infarction**	0.79419	0.910020	0.943200	0.951600
**Electronic pacemaker**	0.98888	0.993120	0.995560	0.994680
**UHN-ECG reduced LVEF**				
**LVEF≤30%**	-	0.923840	0.932350	0.934230
**LVEF≤35%**	-	0.912320	0.922110	0.925440
**LVEF≤40%**	-	0.903190	0.927620	0.931130
**LVEF≤50%**	-	0.852490	0.879450	0.885500

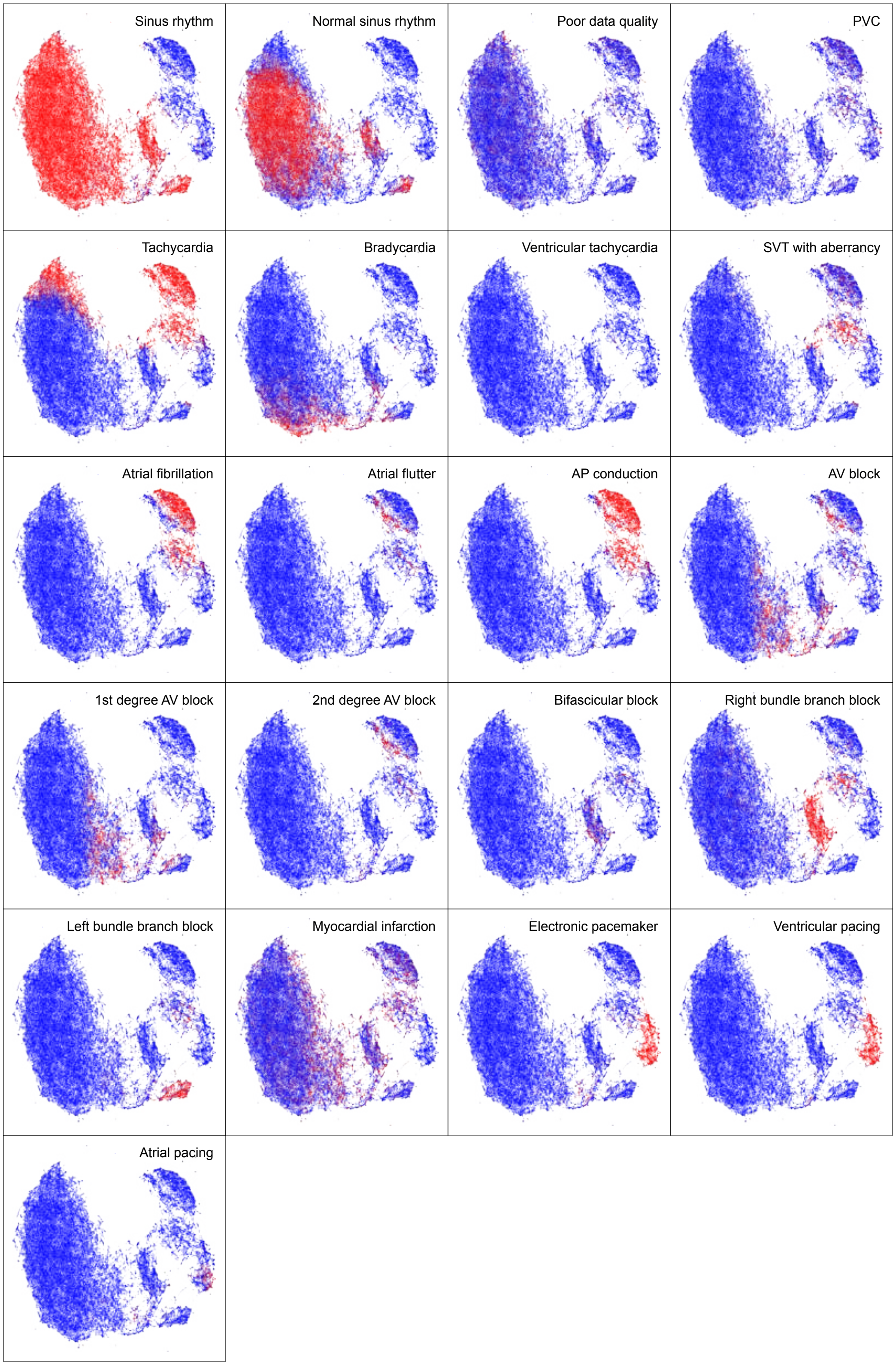

Fig. S2. Label-specific pretrained latent space UMAPs. Label-specific UMAP visualizations of pretrained ECG-FM global representations from ECGs in the UHN-ECG dataset. Each subplot shows a different label from the UHN-ECG interpretation task, where positive samples are indicated in red.

Table S7. UHN-ECG interpretation test results for *Full*. Label-specific test performances on our pretrained, full-finetuned model using the full-scale training dataset.

Label	AUROC	AUPRC	Recall	Precision	F1	Specificity	NPV	Accuracy
**Sinus rhythm**	0.992	0.998	0.990	0.979	0.984	0.899	0.948	0.974
**Normal sinus rhythm**	0.978	0.970	0.943	0.922	0.932	0.928	0.948	0.935
**Poor data quality**	0.900	0.658	0.704	0.529	0.604	0.911	0.956	0.886
**PVC**	0.944	0.752	0.803	0.486	0.605	0.940	0.986	0.931
**Tachycardia**	0.996	0.984	0.968	0.912	0.939	0.975	0.991	0.973
**Ventricular tachycardia**	0.992	0.453	0.755	0.356	0.484	0.999	1.000	0.999
**SVT with aberrancy**	0.983	0.807	0.850	0.575	0.686	0.968	0.992	0.962
**Atrial fibrillation**	0.996	0.968	0.957	0.814	0.880	0.980	0.996	0.978
**Atrial flutter**	0.986	0.761	0.846	0.541	0.659	0.984	0.997	0.981
**Bradycardia**	0.995	0.970	0.935	0.889	0.911	0.983	0.991	0.977
**Accessory pathway conduction**	0.995	0.973	0.930	0.918	0.924	0.990	0.992	0.984
**AV block**	0.987	0.908	0.866	0.802	0.833	0.978	0.986	0.967
**1st degree AV block**	0.992	0.920	0.856	0.859	0.857	0.990	0.989	0.980
**2nd degree AV block**	0.985	0.719	0.727	0.641	0.681	0.991	0.994	0.985
**Bifascicular block**	0.990	0.688	0.678	0.616	0.645	0.992	0.994	0.986
**RBBB**	0.991	0.939	0.907	0.814	0.858	0.975	0.989	0.968
**LBBB**	0.993	0.864	0.807	0.731	0.767	0.990	0.993	0.983
**Myocardial infarction**	0.939	0.820	0.810	0.667	0.732	0.900	0.950	0.882
**Electronic pacemaker**	0.983	0.931	0.969	0.235	0.379	0.799	0.998	0.810
**Ventricular pacing**	0.997	0.969	0.947	0.948	0.947	0.998	0.998	0.995
**Atrial pacing**	0.996	0.920	0.939	0.788	0.857	0.996	0.999	0.995

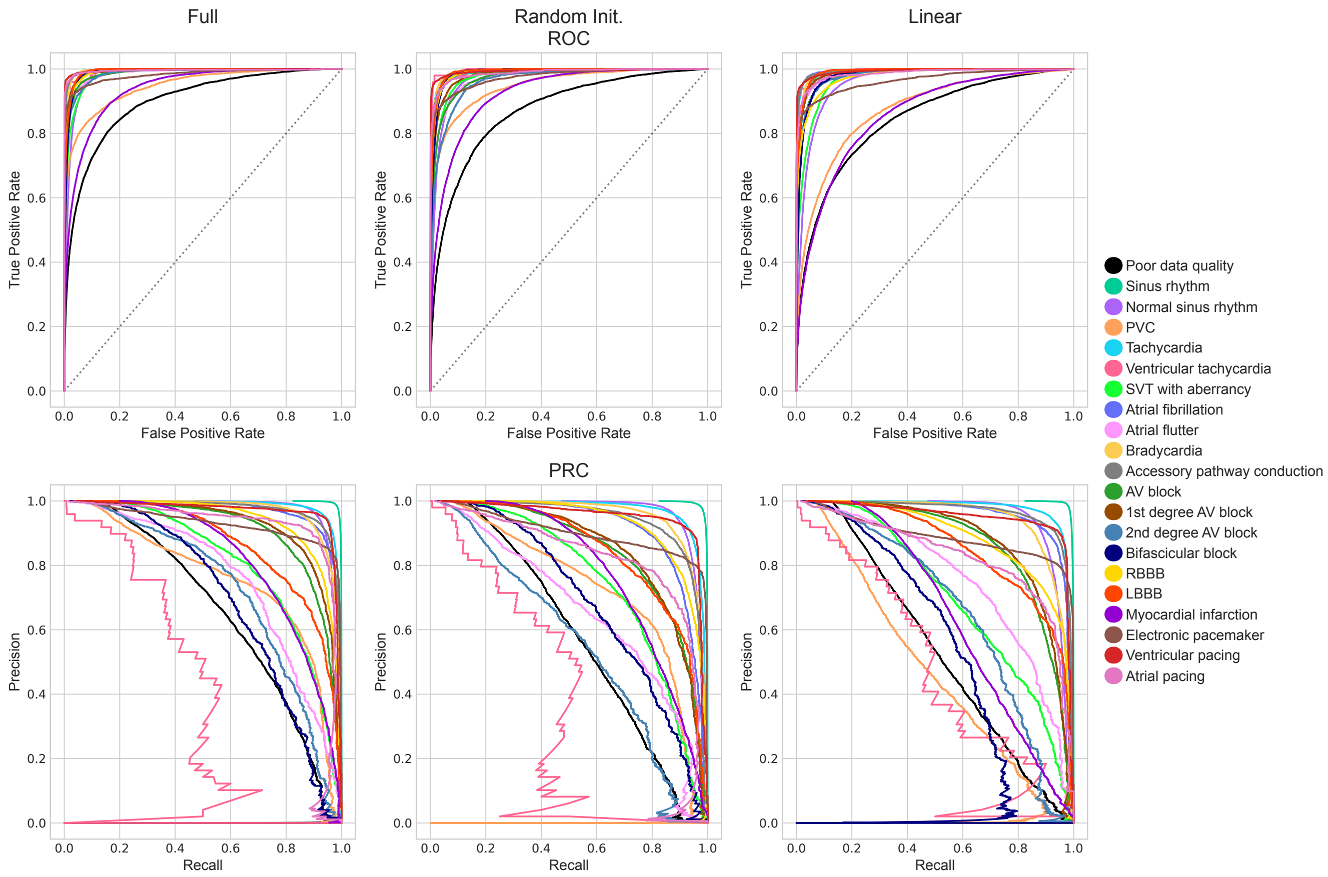

Fig. S3. UHN-ECG interpretation task test performance curves.

Table S8. MIMIC-IV-ECG machine read test results for *Full*. Label-specific test performances on our pre-trained, full-finetuned model using the full-scale training dataset.

Label	AUROC	AUPRC	Recall	Precision	F1	Specificity	NPV	Accuracy
**Poor data quality**	0.783	0.214	0.396	0.176	0.243	0.951	0.984	0.937
**Sinus rhythm**	0.968	0.991	0.959	0.967	0.963	0.863	0.832	0.940
**PVC**	0.908	0.610	0.721	0.423	0.533	0.929	0.979	0.915
**Tachycardia**	0.968	0.910	0.903	0.858	0.880	0.960	0.974	0.948
**Ventricular tachycardia**	0.948	0.186	0.414	0.144	0.214	0.998	0.999	0.997
**SVT with aberrancy**	0.961	0.673	0.685	0.613	0.647	0.982	0.987	0.970
**Atrial fibrillation**	0.976	0.883	0.897	0.731	0.805	0.962	0.988	0.955
**Atrial flutter**	0.931	0.471	0.580	0.428	0.493	0.986	0.992	0.978
**Bradycardia**	0.965	0.860	0.848	0.819	0.833	0.972	0.977	0.955
**Accessory pathway conduction**	0.972	0.888	0.869	0.800	0.833	0.970	0.982	0.958
**AV block**	0.971	0.833	0.856	0.681	0.759	0.965	0.987	0.956
**1st degree AV block**	0.978	0.849	0.859	0.761	0.807	0.978	0.988	0.969
**Bifascicular block**	0.987	0.800	0.783	0.739	0.760	0.992	0.993	0.985
**RBBB**	0.987	0.909	0.879	0.821	0.849	0.983	0.989	0.975
**LBBB**	0.986	0.836	0.848	0.687	0.759	0.985	0.994	0.979
**Myocardial infarction**	0.928	0.820	0.812	0.661	0.729	0.879	0.942	0.864
**Electronic pacemaker**	0.976	0.812	0.863	0.763	0.810	0.988	0.994	0.983

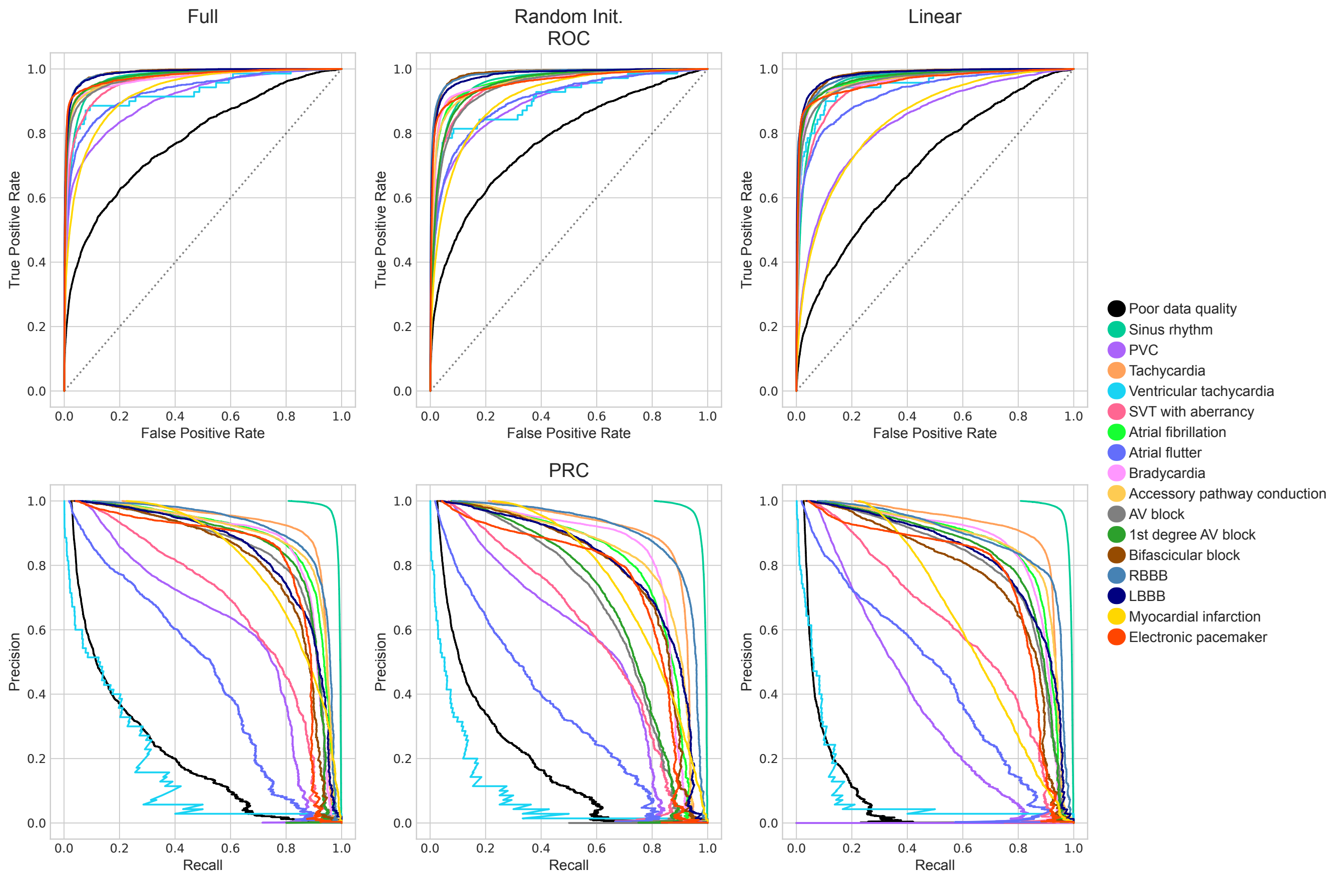

Fig. S4. MIMIC-IV-ECG machine reads task test performance curves.

Table S9. UHN-ECG reduced LVEF test results. Label-specific test performances across all experiment suites using the full-scale training dataset.

Label	Experiment	AUROC	AUPRC	Recall	Precision	F1	Specificity	NPV	Accuracy
**LVEF≤30%**	**Full**	0.918	0.674	0.678	0.594	0.633	0.914	0.938	0.876
	**Linear**	0.905	0.652	0.703	0.565	0.626	0.899	0.942	0.868
	**Random Init.**	0.875	0.585	0.647	0.490	0.558	0.874	0.930	0.839
	**Nejedly**	0.891	0.623	0.691	0.522	0.595	0.882	0.939	0.852
	**SE-WRN**	0.895	0.624	0.688	0.518	0.591	0.880	0.938	0.850
**LVEF≤35%**	**Full**	0.921	0.754	0.758	0.659	0.705	0.892	0.931	0.864
	**Linear**	0.905	0.723	0.775	0.602	0.678	0.860	0.933	0.842
	**Random Init.**	0.876	0.660	0.723	0.544	0.621	0.834	0.917	0.810
	**Nejedly**	0.893	0.696	0.755	0.586	0.660	0.854	0.927	0.832
	**SE-WRN**	0.896	0.705	0.761	0.588	0.664	0.854	0.929	0.834
**LVEF≤40%**	**Full**	0.929	0.825	0.880	0.643	0.743	0.824	0.950	0.839
	**Linear**	0.914	0.785	0.889	0.596	0.714	0.784	0.951	0.811
	**Random Init.**	0.879	0.713	0.870	0.533	0.661	0.726	0.939	0.764
	**Nejedly**	0.896	0.754	0.865	0.576	0.692	0.771	0.941	0.796
	**SE-WRN**	0.900	0.760	0.867	0.583	0.697	0.777	0.942	0.801
**LVEF≤50%**	**Full**	0.911	0.878	0.913	0.666	0.770	0.701	0.925	0.785
	**Linear**	0.889	0.849	0.894	0.646	0.750	0.681	0.908	0.765
	**Random Init.**	0.860	0.800	0.910	0.590	0.716	0.588	0.909	0.715
	**Nejedly**	0.875	0.827	0.910	0.600	0.723	0.604	0.911	0.725
	**SE-WRN**	0.886	0.843	0.913	0.614	0.734	0.626	0.917	0.739

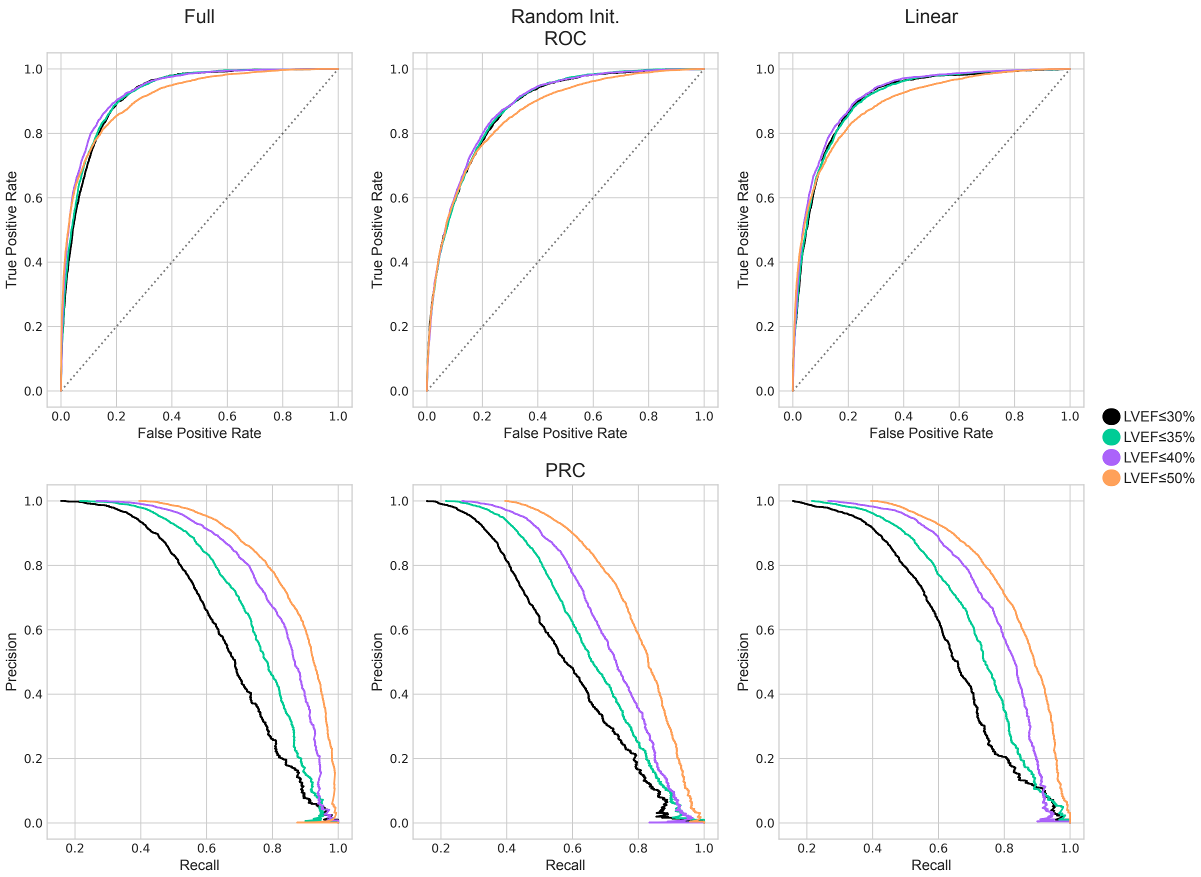

Fig. S5. UHN-ECG reduced LVEF task test performance curves.
